# A novel Cbx1, PurB, and Sp3 complex mediates long-term silencing of tissue- and lineage-specific genes

**DOI:** 10.1016/j.jbc.2022.102053

**Published:** 2022-05-20

**Authors:** Syeda Samara Baksh, Richard E. Pratt, José Gomez, Victor J. Dzau, Conrad P. Hodgkinson

**Affiliations:** 1 Mandel Center for Heart and Vascular Research, and the Duke Cardiovascular Research Center, Duke University Medical Center, Durham, North Carolina, USA; 2 Clinical Pharmacology Division, Department of Medicine, Vanderbilt University Medical Center, Nashville Tennessee, USA

**Keywords:** cardiac development, cardiac muscle, reprogramming, regeneration, fibroblast, cardiomyocyte, transcription repressor, Cas9, CRISPR-associated protein 9, cDNA, complementary DNA, ChIP, chromatin immunoprecipitation, FBS, fetal bovine serum, GO, Gene Ontology, gRNA, guide RNA, H3K27me3, trimethylated histone-H3 lysine-27, iPS, inducible pluripotent stem, MNase, micrococcal nuclease, NEB, New England BioLabs, qPCR, quantitative PCR

## Abstract

miRNA-based cellular fate reprogramming offers an opportunity to investigate the mechanisms of long-term gene silencing. To further understand how genes are silenced in a tissue-specific manner, we leveraged our miRNA-based method of reprogramming fibroblasts into cardiomyocytes. Through screening approaches, we identified three proteins that were downregulated during reprogramming of fibroblasts into cardiomyocytes: heterochromatin protein Cbx1, transcriptional activator protein PurB, and transcription factor Sp3. We show that knockdown of Cbx1, PurB, and Sp3 was sufficient to induce cardiomyocyte gene expression in fibroblasts. Similarly, gene editing to ablate Cbx1, PurB, and Sp3 expression induced fibroblasts to convert into cardiomyocytes *in vivo*. Furthermore, high-throughput DNA sequencing and coimmunoprecipitation experiments indicated that Cbx1, PurB, and Sp3 also bound together as a complex and were necessary to localize nucleosomes to cardiomyocyte genes on the chromosome. Finally, we found that the expression of these genes led to nucleosome modification *via* H3K27me3 (trimethylated histone-H3 lysine-27) deposition through an interaction with the polycomb repressive PRC2 complex. In summary, we conclude that Cbx1, PurB, and Sp3 control cell fate by actively repressing lineage-specific genes.

Cellular reprogramming has the potential to transform regenerative medicine. The standard approach for cellular reprogramming is to use specific combinations of transcription factors, pharmacological agents, or miRNAs (1, 2, 3). With respect to miRNA-based reprogramming, we were interested to identify miRNAs that would reprogram fibroblasts into cardiomyocytes. Through a screening approach, we found that fibroblasts could be converted into cardiomyocytes *via* a combination of four miRNAs (miR-1, miR-133, miR-208, and miR-499), which we called miR combo (4). Importantly, miR combo directly reprograms fibroblasts into cardiomyocytes without the cells passing through an intermediate stem cell state (4). Since our initial discovery, we have gone on to demonstrate that miR combo improves cardiac function in heart injury models (5). Moreover, we have also found that reprogramming *via* miR combo utilizes immunity and epigenetic pathways (6, 7). In addition to reprogramming fibroblasts to cardiomyocytes, miRNAs have also been used to reprogram cells to pluripotency (8) as well as to neurons (9). Compared with transcription factor–based approaches, miRNA-based reprogramming is fundamentally different as it involves the downregulation of a large number of mRNAs (5, 10, 11). The implication of miRNA-based reprogramming is that cells maintain their identity *via* repressive mechanisms (5, 10, 11). Indeed, the majority of genes in eukaryotes are typically silent. Genes that are active during embryogenesis are quickly silenced and remain so throughout development. Moreover, tissue-specific genes are mostly silent at an early stage of development and remain so in most cell types, only undergoing reactivation in their tissues of expression. While there has been considerable focus on gene activation, far less attention has been paid to understanding long-term gene silencing (12). It is believed that long-term gene silencing involves sequence-dependent repression factors, DNA methylation, timing of replication, and histone modifications (12, 13, 14, 15). It is unknown if these mechanisms work independently of each other or in combination. Similarly, it is also unclear how essentially random processes such as DNA methylations or histone modifications are localized to specific genes. For example, the enzymes that modify histones lack any intrinsic ability to distinguish between histones on different genes. Despite this, silencing histone modifications are highly localized. Consequently, understanding the mechanisms underpinning miRNA-based reprogramming is likely to provide important insights into how genes are silenced.

We have previously shown that a combination of four miRNAs (miR-1, miR-133, miR-208, and miR-499) called miR combo reprograms fibroblasts into cardiomyocytes (4, 5, 6, 7, 10, 11, 16, 17). We wanted to leverage our miRNA-based reprogramming method to understand how tissue-specific genes are silenced. Using an siRNA-based screening approach, we identified three miR combo targets: Cbx1, PurB, and Sp3. Gene editing to ablate Cbx1, PurB, and Sp3 expression was sufficient to convert fibroblasts into cardiomyocytes *in vivo*. Chromatin immunoprecipitation (ChIP)-Seq, micrococcal nuclease (MNase)-Seq, and coimmunoprecipitation studies indicated that Cbx1, PurB, and Sp3 bound together as a complex; localized nucleosomes to cardiomyocyte genes; as well as leading to trimethylated histone-H3 lysine-27 (H3K27me3) modification of these nucleosomes *via* an interaction with the PRC2 complex.

## Results

### Identifying potential repressors of the cardiomyocyte phenotype

**Figure 1:**
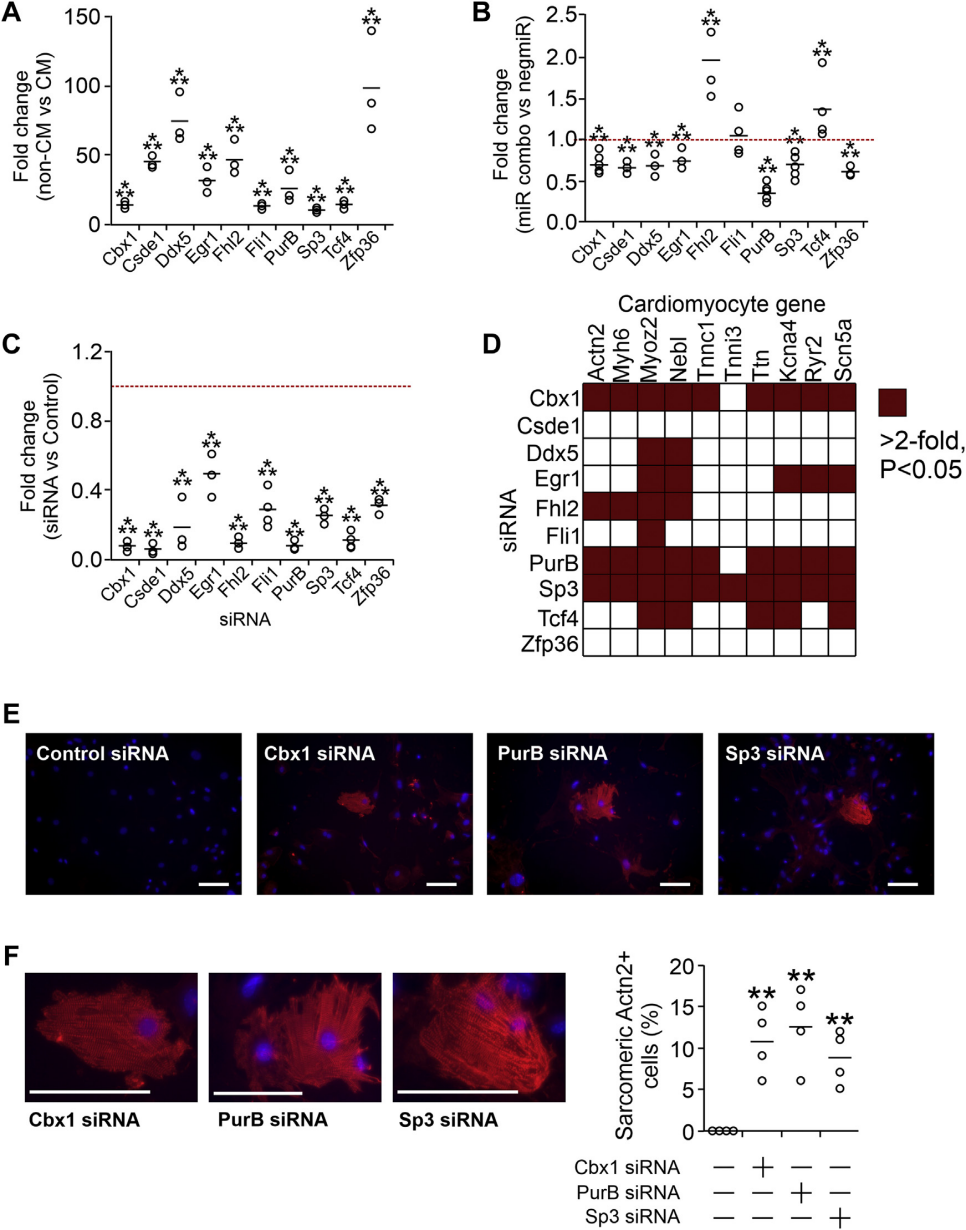
**Cbx1, PurB, and Sp3 are repressors of cardiomyocyte-specific genes.***A*, RNA-Seq read counts from the study by Hodgkinson *et al.* (18) were analyzed for the mRNA levels of the indicated genes in freshly isolated cardiomyocytes and noncardiomyocytes. Individual data points (*open circles*) and mean (*horizontal bar*) are shown. One-way ANOVA with Bonferroni post hoc tests was used to determine significance; ∗∗∗*p* < 0.001. N = 3. *B*, cardiac fibroblasts were transfected with the direct cardiac reprogramming cocktail miR combo. A nontargeting miRNA (negmiR) was used as a control. Four days after transfection, expression of the indicated transcription factors was determined by quantitative PCR (qPCR). Expression values were normalized to negmiR. N = 5. Individual data points (*open circles*) and mean (*horizontal bar*) are shown. One-way ANOVA with Bonferroni post hoc tests was used to determine significance; ∗∗∗*p* < 0.001. *C*, cardiac fibroblasts were transfected with siRNA targeting an individual putative repressor. A nontargeting siRNA was used as a control. After 4 days, expression was determined by qPCR. Expression values were normalized to the control siRNA. N = 3. Individual data points (*open circles*) and mean (*horizontal bar*) are shown. One-way ANOVA with Bonferroni post hoc tests was used to determine significance; ∗∗∗*p* < 0.001. *D*, cardiac fibroblasts were transfected with siRNA targeting an individual putative repressor. A nontargeting siRNA was used as a control. After 14 days, expression of the indicated cardiomyocyte-specific genes was determined by qPCR. Expression values were normalized to the control siRNA. The heatmap summarizes the results of ten cardiomyocyte-specific genes. Increased expression of greater than twofold and with a significance <0.05 is shown in *red*. N = 3 to 5. One-way ANOVA with Bonferroni post hoc tests was used to determine significance; ∗*p* < 0.05. *E*, cardiac fibroblasts were transfected with siRNA targeting an individual putative repressor. A nontargeting siRNA was used as a control. After 14 days, the cells were incubated with antibodies to the cardiomyocyte-specific protein Actn2. Representative images are shown. N = 4. The scale bar represents 50 microns. *F*, higher resolution images of the cells shown in *E* are shown to display sarcomeres. The scale bar represents 50 microns. N = 4. Quantification of the percentage of Actn2+ cells displaying sarcomeres. Individual data points (*open circles*) and mean (*horizontal bar*) are shown. One-way ANOVA with Bonferroni post hoc tests was used to determine significance; ∗∗*p* < 0.01.

We have previously demonstrated that a combination of four miRNAs (miR-1, miR-133, miR-208, and miR-499), which we call miR combo, reprograms fibroblasts into cardiomyocytes (4, 5). Considering that miRNAs initiate the degradation of their mRNA targets, this implies that repressors of cardiomyocyte genes should be found among the targets of miR combo. To identify potential targets, we analyzed our recent RNA-Seq study that compared various cardiac populations in the mouse heart (18). Through this approach, we found that when compared with undifferentiated cells, cardiomyocytes were depleted for ∼80 transcription factors and RNA-binding proteins. The list was filtered by removing proteins previously implicated in the differentiation to noncardiomyocyte lineages such as blood vessels or neurons. After filtering, ten potential candidates were identified: Cbx1, Csde1, Ddx5, Egr1, Fhl2, Fli1, PurB, Sp3, Tcf4, and Zfp36 (Fig. 1*A*). Of these ten candidates, Cbx1, Csde1, Ddx5, Egr1, PurB, Sp3, and Zfp36 were found to be targets of miR combo (Fig. 1*B*).

To evaluate the role of these ten candidates in regulating the expression of cardiomyocyte-specific genes, knockdown experiments were performed. Knockdown of each candidate was robust (Fig. 1*C*). Following knockdown of the candidate repressor in fibroblasts, expression levels of cardiomyocyte-specific genes were measured. With the exception of Csde1, knockdown of each candidate repressor induced the expression of at least one cardiomyocyte-specific gene (Fig. 1*D*). Of the ten potential repressors, knockdown of Cbx1, PurB, or Sp3 increased the expression of >90% of the measured cardiomyocyte-specific genes (Fig. 1*D*, representative graphs in Fig. S1). As Cbx1, PurB, and Sp3 appeared to be universal regulators of the cardiomyocyte phenotype, they were studied further.

Knockdown of Cbx1, PurB, or Sp3 was sufficient to reprogram fibroblasts into cardiomyocyte-like cells as evidenced by expression of the cardiomyocyte protein Actn2 (Fig. 1*E*) as well as sarcomere formation (Fig. 1*F*).

In contrast to cardiac fibroblasts, knockdown of Cbx1, PurB, or Sp3 did not induce cardiomyocyte gene expression in lung or tail-tip fibroblasts (Fig. S2).

**Figure 2:**
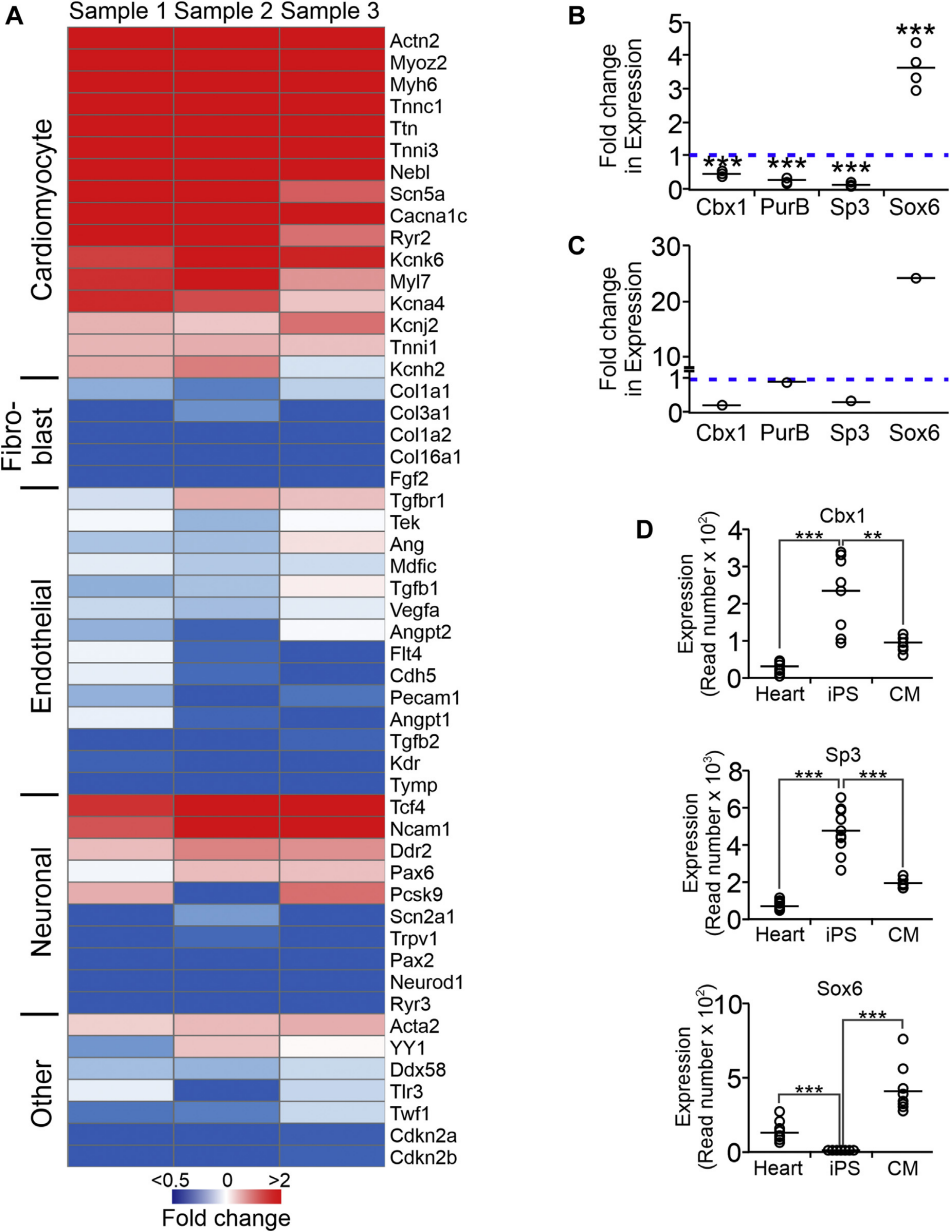
**Loss of Cbx1, PurB, and Sp3 expression is associated with cardiomyocyte differentiation.***A*, cardiac fibroblasts were transfected with either a control nontargeting siRNA or a pool of siRNAs targeting Cbx1, PurB, and Sp3. After 14 days, expression of the indicated cardiomyocyte, fibroblast, endothelial, and neuronal-specific genes was determined by quantitative PCR (qPCR). For completeness, a number of general genes were included. Expression values were normalized to the control siRNA. N = 3. *B*, RNA was extracted from iPS cells and iPS-derived cardiomyocytes. Expression of the indicated genes was determined by qPCR, and expression values in iPS-derived cardiomyocytes were normalized to iPS cells. N = 4. Individual data points (*open circles*) and mean (*horizontal bar*) are shown. One-way ANOVA with Bonferroni post hoc tests was used to determine significance; ∗∗∗*p* < 0.01. *C*, RNA-Seq of iPS differentiation into cardiomyocytes from the study by Churko *et al.* (20). Expression values of the indicated genes in iPS-derived cardiomyocytes were normalized to iPS cells. N = 1. *D*, RNA-Seq from the study by Pavlovic *et al.* (21). Cardiac tissue was isolated from 12 individuals and used to generate iPS cells. These iPS cells were then differentiated into cardiomyocytes. RNA-Seq was performed with the source cardiac tissue; undifferentiated iPS cells; as well as iPS-derived cardiomyocytes. N = 12. Read number is shown. Individual data points (*open circles*) and mean (*horizontal bar*) are shown. One-way ANOVA with Bonferroni post hoc tests was used to determine significance; ∗∗*p* < 0.01 and ∗∗∗*p* < 0.001. iPS, inducible pluripotent stem.

To further investigate the role of Cbx1, PurB, and Sp3 as repressors, we investigated the effect of their knockdown on fibroblast, endothelial, and neuronal specific gene expression. As expected, knockdown of Cbx1, PurB, and Sp3 strongly induced cardiomyocyte-specific gene expression. In contrast, loss of Cbx1, PurB, and Sp3 reduced the expression of fibroblast-specific genes (Fig. 2*A*). This result suggested that the fibroblasts were indeed exiting the fibroblast phenotype. Similarly, loss of Cbx1, PurB, and Sp3 reduced endothelial gene expression (Fig. 2*A*). Generally, neuronal markers were not expressed in fibroblasts or expressed at low levels. Despite the apparent induction of a few neuronal markers, loss of Cbx1, PurB, and Sp3 generally reduced neuronal gene expression (Fig. 2*A*).

### Loss of Cbx1, PurB, and Sp3 expression is associated with cardiomyocyte development

If Cbx1, PurB, and Sp3 play an important role in repressing cardiomyocyte-specific genes, their expression should decrease during cardiomyocyte development. In support of this hypothesis, we found that inducible pluripotent stem (iPS) cell differentiation to cardiomyocytes was associated with a significant decrease in the expression of Cbx1, PurB, and Sp3 (Fig. 2*B*). To demonstrate that the loss of Cbx1, PurB, and Sp3 was not because of a general loss of expression, we also measured the expression of Sox6; a transcription factor expressed during cardiomyocyte differentiation (19). As expected, Sox6 expression increased during iPS differentiation to cardiomyocytes (Fig. 2*B*).

To verify these results, we utilized publically available RNA-Seq data. We chose two separate RNA-Seq studies that measured mRNA changes in human iPS cells undergoing differentiation into cardiomyocytes. Churko *et al.* (20) provided an averaged mRNA read count (ten technical replicates) of a single human iPS cell line at various time points during cardiomyocyte differentiation. In contrast, Pavlovic *et al.* (21) provided mRNA read count data from 12 individual human iPS lines. In addition, the Pavlovic study also provided the mRNA read data from the cardiac tissues that were used to generate the individual iPS lines. Analysis of both datasets supported our findings. In the Churko study, Cbx1, PurB, and Sp3 expression were all reduced in iPS-derived cardiomyocytes when compared with iPS cells (Fig. 2*C*). Again, Sox6 expression increased (Fig. 2*C*). PurB levels were not measured in the Pavlovic study; however, in all 12 human iPS cell lines, cardiomyocyte differentiation was associated with significant loss of Cbx1 and Sp3 expression (Fig. 2*D*). Cbx1 and Sp3 expression were similar in iPS-derived cardiomyocytes and the heart tissue from which the iPS cells were generated (Fig. 2*D*). In contrast, Sox6 expression increased during iPS cell differentiation to cardiomyocytes in all 12 lines (Fig. 2*D*).

### Cbx1, PurB, and Sp3 knockout induces fibroblasts to reprogram into cardiomyocytes *in vivo*

**Figure 3:**
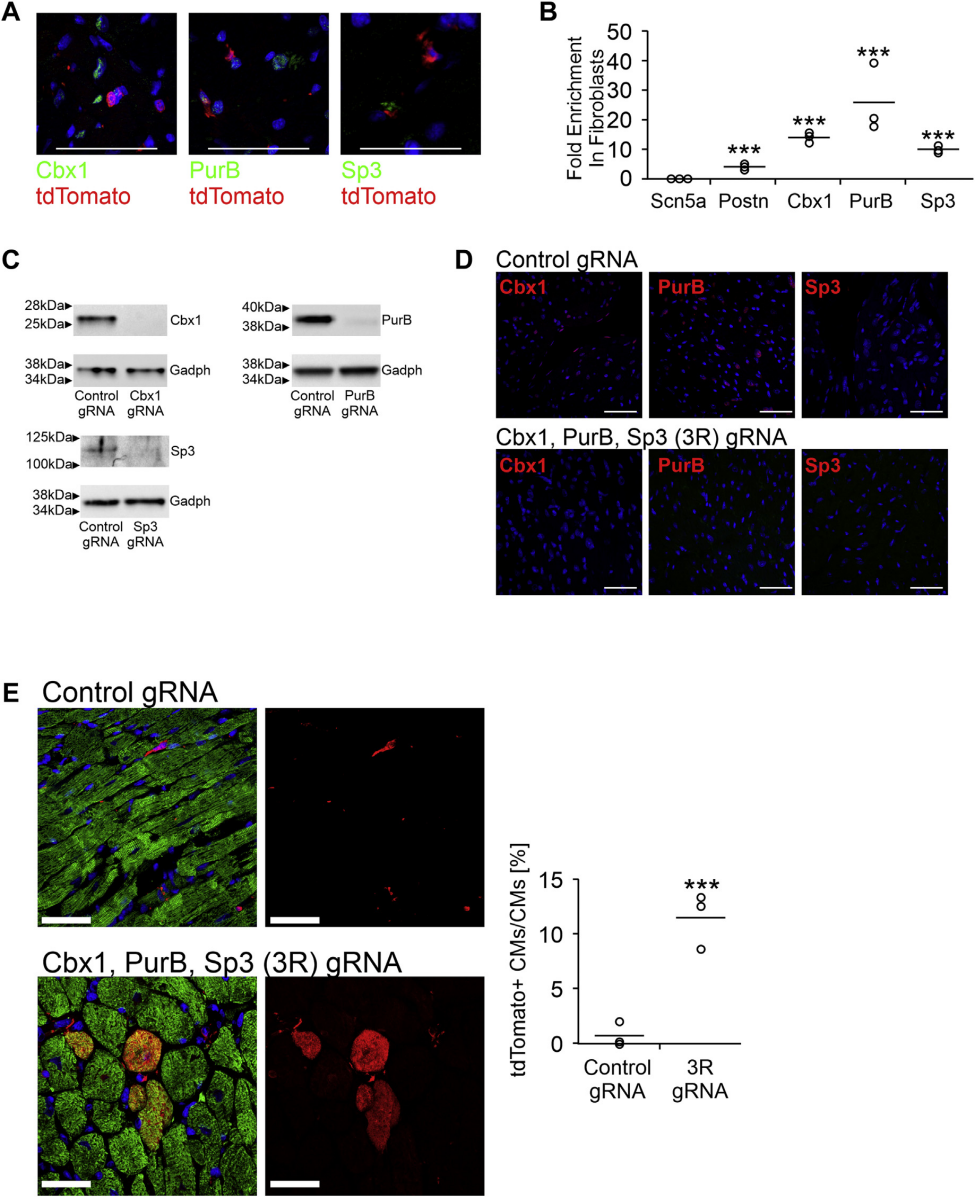
**Ablation of Cbx1, PurB, and Sp3 expression reprograms fibroblasts into cardiomyocytes.***A*, cardiac tissue was isolated from 8-week-old Fsp1-Cre:tdTomato mice. In these mice, Fsp1 fibroblasts are marked permanently with tdTomato. Tissue slices were incubated with tdTomato (*red*) and repressor (*green*) antibodies. Nuclei (*blue*) were stained with 4′,6-diamidino-2-phenylindole (DAPI). Representative images from three individual mice. The scale bar represents 50 microns. *B*, cardiomyocytes and fibroblasts were isolated from 1-day-old C57BL6 mice. RNA was analyzed for the expression of the cardiomyocyte marker Scn5a, the fibroblast marker Postn, as well as the expression of the three repressors. Expression values are shown as a fold enrichment in fibroblasts when compared with cardiomyocytes. N = 3. Individual data points (*open circles*) and mean (*horizontal bar*) are shown. One-way ANOVA with Bonferroni post hoc tests was used to determine significance; ∗∗∗*p* < 0.001. *C*, guide RNAs (gRNAs) for Cbx1, PurB, and Sp3 were cloned into a plasmid containing CRISPR-associated protein 9 (Cas9), and the resulting construct was transfected into cultured cardiac fibroblasts. After 7 days, protein extracts were probed for the presence of Cbx1, PurB, or Sp3. N = 3. Representative blots are shown with the loading control Gapdh. *D*, the Cbx1, PurB, and Sp3 gRNAs were cloned into a lentivirus-generating plasmid containing Cas9. Control nontargeting gRNA was cloned into the same plasmid as a control. Lentiviral particles were isolated and injected into the heart of an 8-week-old C57BL6 mouse. One week after cardiac injection, tissue slices were analyzed for repressor expression (*green*). Nuclei (*blue*) were visualized *via* DAPI. The scale bar represents 50 microns. Representative images from three individual mice. *E*, the Cbx1, PurB, and Sp3 gRNAs were cloned into a lentivirus-generating plasmid containing Cas9. Lentiviral particles were injected into the hearts of fibroblast lineage-tracing mouse Fsp1-Cre:tdTomato. In this model, fibroblasts and their progeny are permanently labeled with the fluorescent protein tdTomato. Two months after injection, heart sections within 500 microns of the injection site were incubated with tdTomato and cardiac troponin-T (cardiomyocyte-specific marker) antibodies. Representative images are shown. The scale bar represents 50 microns. N = 3 per group. The number of cardiomyocytes derived from the reprogramming of fibroblasts (tdTomato+ cardiac troponin-T+) is expressed as a percentage of the total cardiomyocyte (cardiac troponin-T+) population. A two-tailed *t* test was used to determine significance between the two groups; ∗∗∗*p* < 0.001.

We wanted to determine if Cbx1, PurB, and Sp3 also repressed cardiomyocyte genes in *in vivo* fibroblasts. *In vivo*, expression of the three proteins was found to be localized to fibroblasts and absent in cardiomyocytes (Fig. 3, *A* and *B*). CRISPR–CRISPR-associated protein 9 (Cas9) gene editing was employed to ablate Cbx1, PurB, and Sp3 expression *in vivo*. To identify functional guide RNAs (gRNAs), 2 to 3 gRNAs for the first exon of Cbx1, PurB, or Sp3 were introduced individually into cultured cardiac fibroblasts along with Cas9. Immunoblotting indicated that the gRNAs were effective in ablating Cbx1, PurB, and Sp3 expression (Fig. 3*C*). To test efficacy of knockout *in vivo*, the gRNAs for the three proteins as well as Cas9 were subsequently packaged into lentivirus particles and injected into the mouse heart. Seven days later, cardiac tissue was analyzed for the expression of Cbx1, PurB, and Sp3. In control cardiac tissue, Cbx1, PurB, and Sp3 expression was robust and localized to the nucleus (Fig. 3*D*). However, expression of the three proteins was absent in cardiac tissue isolated from mice receiving the repressor gRNAs (Fig. 3*D*). Having demonstrated the efficacy of the approach, control and repressor targeting gRNAs were delivered into the hearts of Fsp1-Cre:tdTomato fibroblast lineage-tracing mice. In these Fsp1-Cre:tdTomato fibroblast lineage-tracing mice, fibroblasts are permanently marked with tdTomato (4). In control mice, injected with lentiviruses containing Cas9 and nontargeting gRNAs, there were no tdTomato+ cardiomyocytes; indicating that tdTomato+ fibroblasts do not normally differentiate into cardiomyocytes (Fig. 3*E*). In contrast, following the ablation of Cbx1, PurB, and Sp3 expression, ∼10% of cardiomyocytes in the vicinity of the injection site were tdTomato+; indicating fibroblast conversion into cardiomyocyte-like cells (Fig. 3*E*).

### Cbx1, PurB, and Sp3 bind specifically to cardiomyocyte-specific genes in fibroblasts

**Figure 4:**
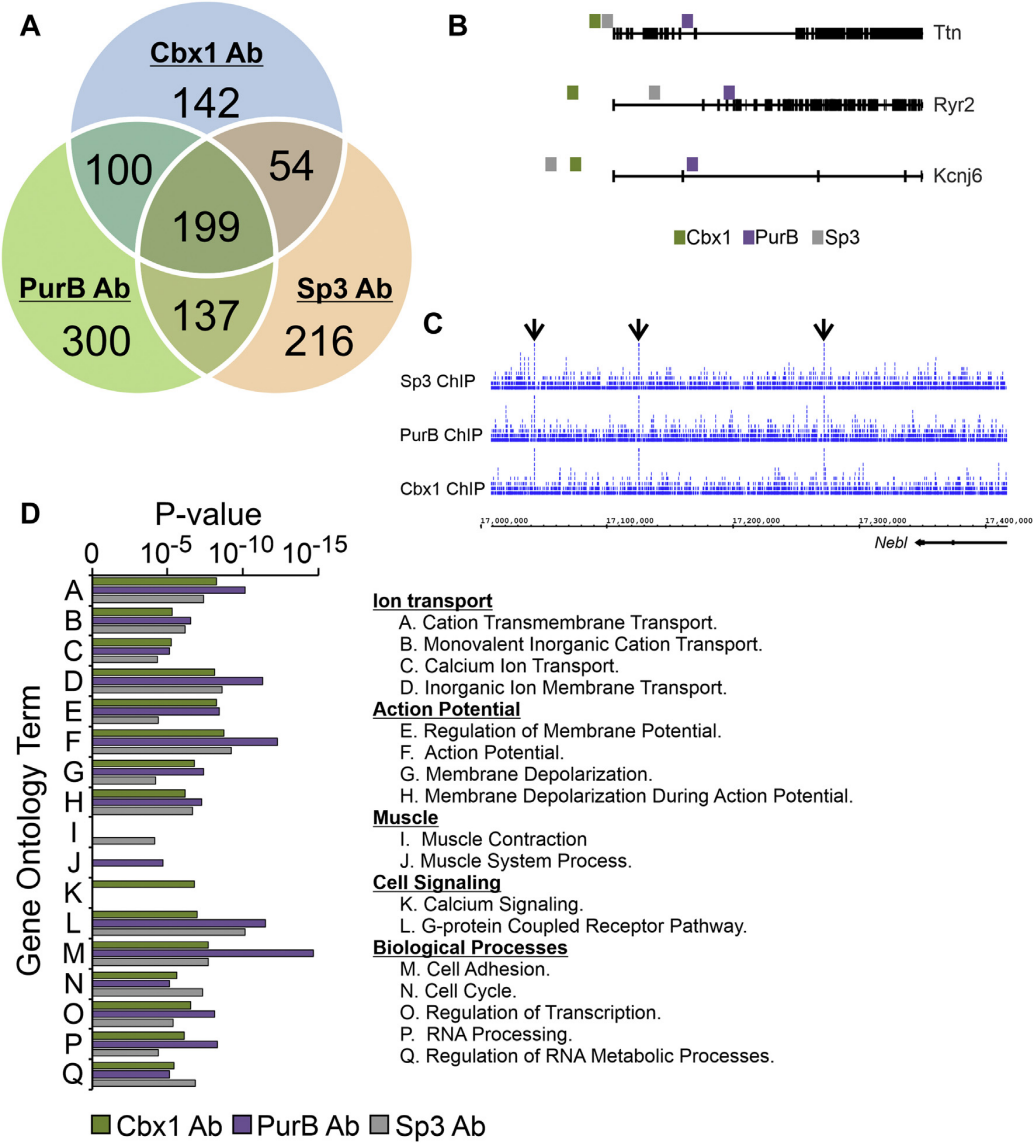
**Cbx1, PurB, and Sp3 bind to cardiomyocyte-specific genes in fibroblasts.***A*, chromatin derived from cardiac fibroblasts was incubated with antibodies for Cbx1, PurB, or Sp3. An isotype antibody was used as a control. Immunoprecipitated DNA was analyzed by high-throughput sequencing. Bioinformatic approaches were used to determine Cbx1-, PurB-, and Sp3-binding sites. The Venn diagram details the number of genes with Cbx1-, PurB-, and Sp3-binding sites. *B*, Cbx1-, PurB-, and Sp3-binding sites in the cardiomyocyte-specific genes Ttn, Ryr2, and Kcnj6. *C*, Cbx1-, PurB-, and Sp3-binding peaks in the *Nebl* gene. *D*, Gene Ontology analysis of the genes to which Cbx1, PurB, and Sp3 were bound.

To understand the mechanism by which these repressors actively repress the cardiomyocyte phenotype in fibroblasts, we first employed ChIP-Seq. Chromatin derived from mouse cardiac fibroblasts was incubated with Cbx1, PurB, or Sp3 antibodies, and the resulting immunoprecipitated DNA was sequenced *via* high-throughput sequencing. Analysis of the dataset indicated that Cbx1, PurB, and Sp3 shared many of the same targets (Fig. 4, *A*; see Table S1 for full target gene list). In the cardiomyocyte-specific genes *Ttn*, *Ryr2*, and *Kcnj6*, Cbx1-binding sites were present in the promoter exclusively (Fig. 4*B*). In contrast, PurB-binding sites were only present within the coding sequence (Fig. 4*B*). Sp3-binding sites were found in both promoter and within the coding sequence (Fig. 4*B*).

Gene Ontology (GO) analysis of repressor-bound genes gave further support to notion that Cbx1, PurB, and Sp3 play an important role in regulating the cardiomyocyte phenotype. Significant GO terms included those for cation transport, formation of the action potential, and muscle contraction (Fig. 4*C*; full GO analysis is provided in Tables S2–S4). Additional significant GO terms included those for calcium signaling as well as biological processes including transcription regulation, cell adhesion, and the cell cycle (Fig. 4*C*; full GO analysis is provided in Tables S2–S4).

### Cbx1, PurB, and Sp3 regulate nucleosome architecture

**Figure 5:**
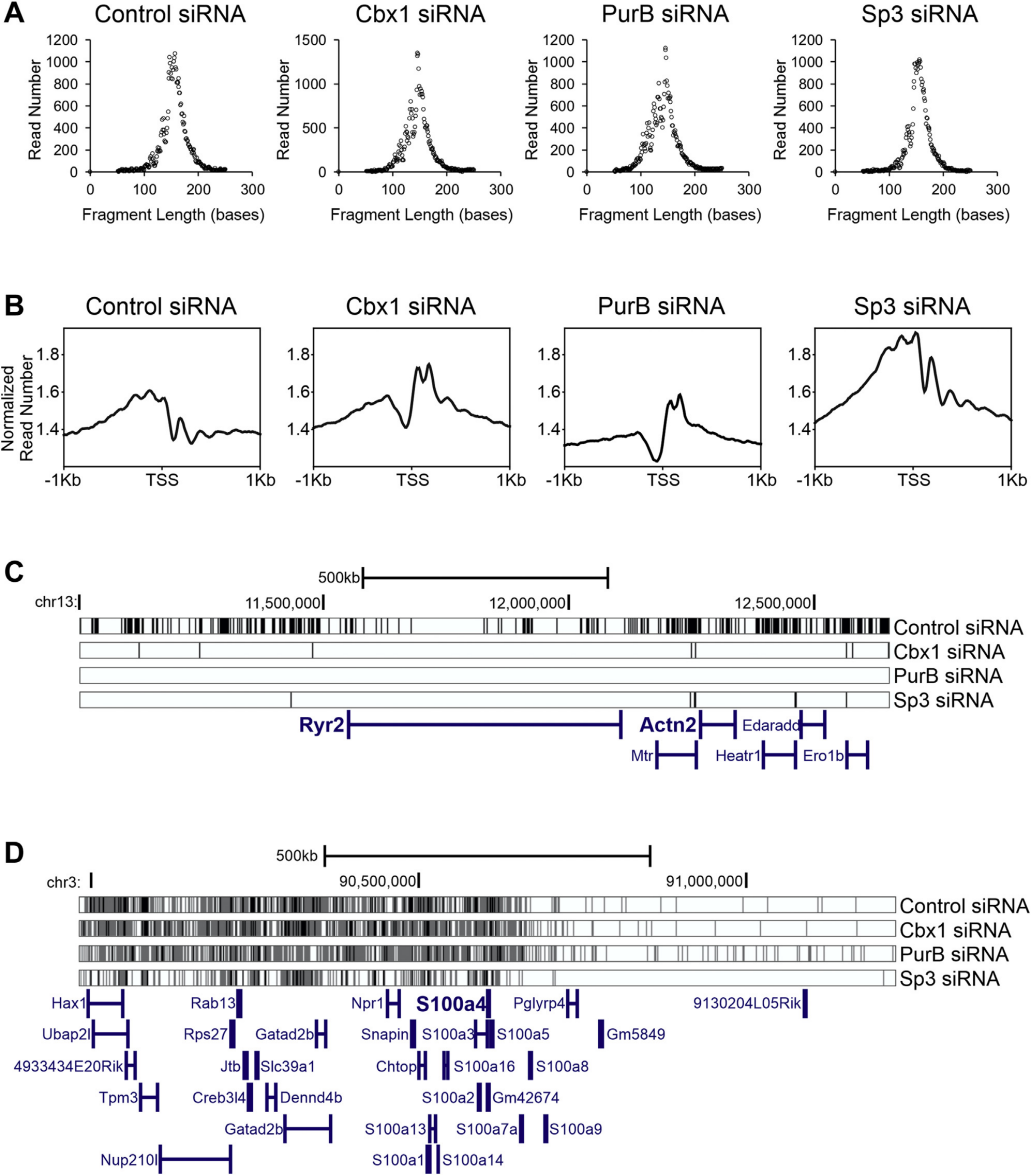
**Cbx1, PurB, and Sp3 regulate chromatin architecture.** Cardiac fibroblasts were transfected with siRNAs targeting Cbx1, PurB, or Sp3. A nontargeting siRNA was used as a control. After 7 days, chromatin was isolated and digested with micrococcal nuclease (MNase). Following MNase digestion, the resulting undigested DNA was submitted for high-throughput sequencing (MNase-Seq) and mapped to the mouse genome. *A*, MNase digestion was optimized to give rise to one nucleosome. Read lengths were analyzed after sequencing and summed. As expected, the majority of read lengths were 1 nucleosome is size (∼150 bp). *B*, MNase accessibility signals around transcription start sites (TSSs). The *y*-axis represents the read number for each 10 bp bin normalized to the effective genome size for the mouse. *C*, nucleosomes (*black bars*) were plotted on the cardiomyocyte-specific genes *Ryr2* and *Actn2*. *D*, nucleosomes in noncardiomyocyte genes.

We hypothesized that Cbx1, PurB, and Sp3 inhibited cardiomyocyte-specific genes in fibroblasts by modifying the nucleosome architecture. To test this hypothesis, we performed MNase-Seq. MNase-Seq is used to map nucleosomes. Nucleosomes are the basic unit of DNA compaction and a fundamental component of chromatin. Cardiac fibroblasts were transfected with either a control nontargeting siRNA or an siRNA that targets Cbx1, PurB, or Sp3. Chromatin was isolated 7 days later and incubated MNase. As shown in Figure 5*A*, MNase digestion conditions were optimized to cut the DNA in lengths of one nucleosome (∼147 bp). The MNase-digested samples were then submitted for high-throughput sequencing. The nucleosome architecture of active eukaryotic genes comprises of a nucleosome-free region just upstream of the transcription start site and an array of regularly spaced nucleosomes over the gene (22). In control cells, this pattern is absent at a genome-wide level (Fig. 5*B*). This suggests that in fibroblasts, the majority of genes are silent. Gene silencing appears to require Cbx1 and PurB as the loss of either protein induced nucleosome-free regions to appear (Fig. 5*B*). Loss of Sp3 differs in that the nucleosome architecture of control cells is retained (Fig. 5*B*). However, seeing as the read density was higher in the Sp3 siRNA-transfected cells, the data suggest that Sp3 plays a role in histone binding (Fig. 5*B*). At the level of individual genes, in control fibroblasts, cardiomyocyte-specific genes such as Ryr2 and Actn2 contain a large number of nucleosomes (Fig. 5*C*). Following knockdown of Cbx1, PurB, or Sp3, these nucleosomes disappear (Fig. 5*C*). In contrast, knockdown of Cbx1, PurB, or Sp3 had no effect on nucleosome patterning in noncardiomyocyte genes (Fig. 5*D*).

### Cbx1, PurB, and Sp3 bind as a complex and interacts with the PRC2 complex

**Figure 6:**
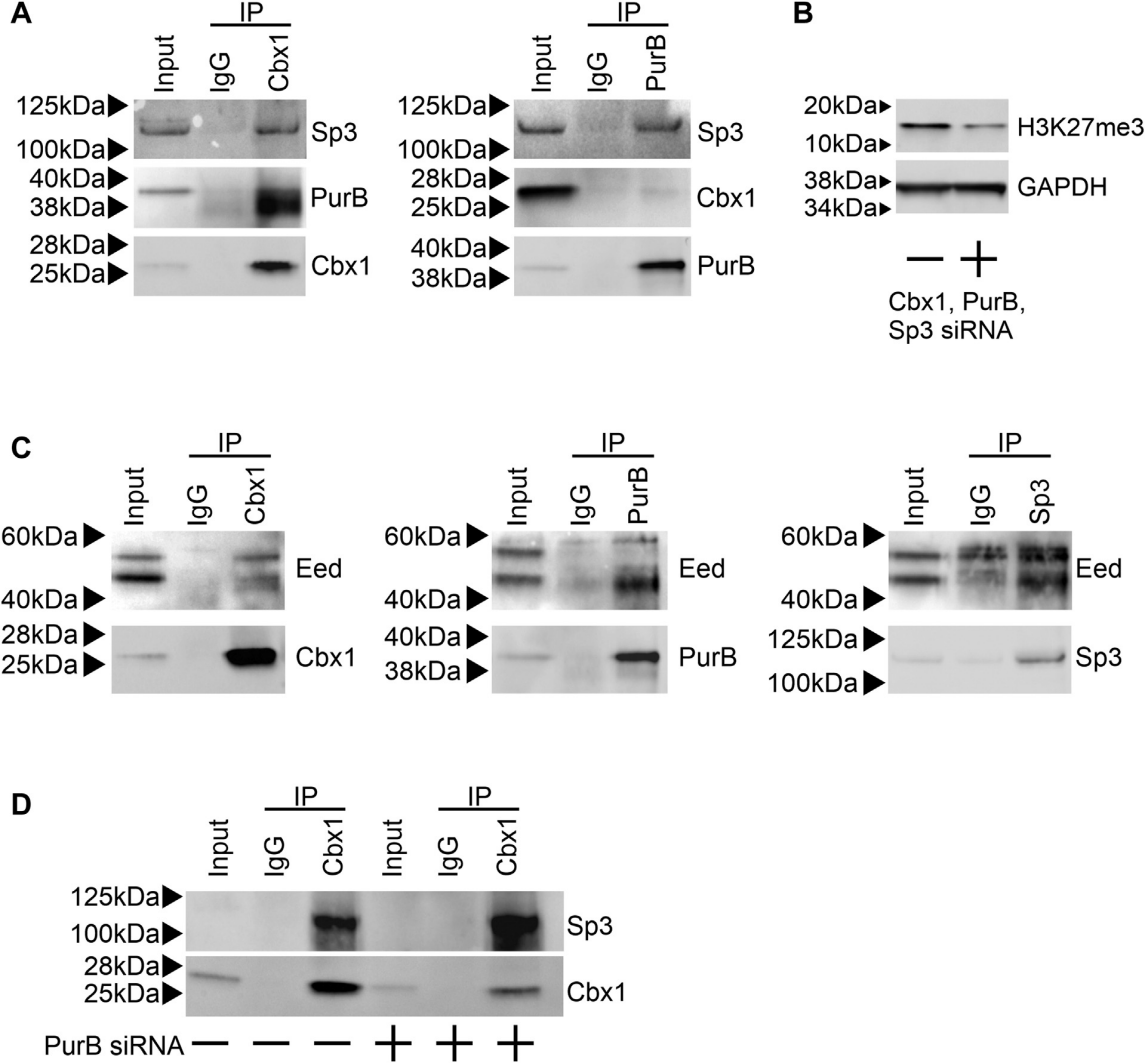
**Cbx1, PurB, and Sp3 regulate the PRC2 complex.***A*, endogenous Cbx1 and PurB was immunoprecipitated from cardiac fibroblast cell lysates. An isotype control antibody was used as a control. Immunoprecipitates were immunoblotted with a Cbx1, PurB, and a Sp3 antibody. The first lane contains cell extract (1/10th immunoprecipitation input). Representative immunoblots are shown from three independent experiments. *B*, cardiac fibroblasts were transfected with siRNAs targeting Cbx1, PurB, and Sp3. A nontargeting siRNA was used as a control. After 4 days, cell lysates were immunoblotted with H3K27me3 and H3 antibodies. Immunoblotting for Gapdh was used as a loading control. Representative immunoblots are shown from four independent experiments. *C*, endogenous Cbx1, PurB, and Sp3 was immunoprecipitated from cardiac fibroblast cell lysates. An isotype control antibody was used as a control. Immunoprecipitates were immunoblotted with an Eed antibody. The first lane contains cell extract (1/10th immunoprecipitation input). Representative immunoblots are shown from three independent experiments. *D*, cardiac fibroblasts were transfected with either a nontargeting control siRNA or a PurB targeting siRNA. After 3 days, endogenous Cbx1 was immunoprecipitated from cell lysates. An isotype control antibody was used as a control. Immunoprecipitates were immunoblotted with a Cbx1 and a Sp3 antibody. The first lane contains cell extract (1/10th immunoprecipitation input). Representative immunoblots are shown from three independent experiments. H3K27me3, trimethylated histone-H3 lysine-27.

The ChIP-Seq data suggested that Cbx1, PurB, and Sp3 may act as a complex. To investigate complex formation, coimmunoprecipitation experiments were performed. Cbx1 immunoprecipitates were found to be highly enriched in Sp3 (Fig. 6*A*). Similarly, Sp3 was also highly enriched in PurB immunoprecipitates (Fig. 6*A*). The data suggest shared protein complexes, with Cbx1–Sp3 and PurB–Sp3 dimers being readily apparent. Cbx1 binding to PurB is somewhat unclear as binding was apparent when the Cbx1 antibody was used but not when the PurB antibody was used instead (Fig. 6*A*). This may be due to steric inhibition between the PurB antibody and the Cbx1 protein.

Having identified binding between the repressors, we wanted to determine how Cbx1, PurB, and Sp3 regulated gene activity. The MNase-Seq data suggested that Cbx1, PurB, and Sp3 were necessary for nucleosome patterning on cardiomyocyte-specific genes especially on gene promoters. Considering their role as repressors, we hypothesized that Cbx1, PurB, and Sp3 were important for the formation of inhibitory nucleosomes. Based on our prior miR combo studies, we further hypothesized that these inhibitory nucleosomes contained H3K27me3. Indeed, the combined knockdown of Cbx1, PurB, and Sp3 was found to reduce H3K27me3 levels (Fig. 6*B*). The formation of H3K27me3 is dependent upon the PRC2 complex, which comprises catalytic (Ezh1, Ezh2) and regulatory (Suz12, Eed) subunits (23). We found that Cbx1, PurB, and Sp3 associated with either one or both of the Eed isoforms (Fig. 6*C*).

The coimmunoprecipitation experiments suggested that Cbx1–Sp3 and PurB–Sp3 are two nonoverlapping complexes. In further support of this notion, knockdown of PurB was found to have no effect on Cbx1–Sp3 complex formation (Fig. 6*D*).

## Discussion

Tissue-specific genes are mostly silent. They are typically silent during early development and remain so in most cell types, only undergoing reactivation in their tissues of expression. A number of mechanisms have been proposed for long-term gene silencing including sequence-dependent repression factors, DNA methylation, timing of replication, and histone modifications (12, 13, 14, 15). It is unknown how these mechanisms relate to each other, whether they are independent or function together. Moreover, it is unclear how silencing DNA or histone modifications are localized to specific genes.

This study suggests that the long-term silencing of tissue-specific genes is regulated by Cbx1, PurB, and Sp3. ChIP-Seq indicated that Cbx1, PurB, and Sp3 were specifically localized to cardiomyocyte genes. Their role appears to gene silencing as genetic ablation of these three proteins *in vivo*, as well as knockdown *in vitro*, was sufficient to induce the expression of cardiomyocyte-specific genes in fibroblasts. Based on the data obtained, it appears that Cbx1, PurB, and Sp3 mediate tissue-specific gene silencing by modifying the nucleosome architecture as well as regulating the deposition of silencing histone modifications. Cardiomyocyte genes in fibroblasts were found to contain a significant number of nucleosomes. A large number of nucleosomes may act to compact the gene and prevent expression. However, following the knockdown of Cbx1, PurB, or Sp3, these nucleosomes were no longer present. Nucleosome-free genes are typically transcriptionally active. The effects on nucleosome patterning were restricted as Cbx1, PurB, or Sp3 knockdown had no effect on nucleosomes in fibroblast genes such as S100a4. How Cbx1, PurB, and Sp3 binding induces nucleosome formation on cardiomyocyte genes is an open question. Cbx1-, PurB-, and Sp3-binding sites within cardiomyocyte genes were distinct and often separated by more than 100 nucleotides. However, coimmunoprecipitation studies suggested shared protein complexes between the three proteins. Complex formation would suggest that Cbx1, PurB, and Sp3 are causing DNA to loop. DNA looping has been invoked as the explanation for the ability of enhancers to increase transcription. It is possible that DNA looping induced by Sp3–Cbx1 and Sp3–PurB dimers acts as a scaffold for nucleosome binding.

The influence of nucleosomes on gene transcription is both simple and complex (24, 25, 26). By virtue of their mere presence, nucleosomes can act as an impediment to transcription by preventing RNA polymerases from moving along the gene. The histone core of the nucleosome can be acetylated or methylated, and the effect of these modifications on gene transcription is more subtle. Depending upon which histone residue is modified, acetylation and methylation can either promote or inhibit gene transcription. Indeed, a hallmark of fibroblast reprogramming to cardiomyocytes is the loss of inhibitory H3K27me3 from cardiomyocyte genes (10). H3K27me3 commonly resides on gene promoters (22). Two lines of evidence suggest that Cbx1, PurB, and Sp3 regulate cardiomyocyte gene activity through mediating H3K27me3 deposition. First, loss of Cbx1 and PurB expression induced histone loss in gene promoters. Second, coimmunoprecipitation studies indicated that Cbx1, PurB, and Sp3 interacted with Eed. Eed is an important component of the PRC2 complex, which mediates H3K27me3 deposition. This suggests that Cbx1, PurB, and Sp3 regulate the activity of the PRC2 complex. Indeed, knockdown of Cbx1, PurB, and Sp3 was found to reduce H3K27me3 levels. While we were able to see reduced H3K27me3 following loss of repressor expression, we were not able to determine if this was specific to cardiomyocyte genes. Future studies are therefore necessary to determine if Cbx1, PurB, and Sp3 regulate H3K27me3 deposition specifically on cardiomyocyte genes. Quantitative PCR (qPCR) analyses proved to be unreliable with apparently specific primers routinely showing multiple bands. Consequently, we plan to carry out these studies by expressing cardiomyocyte and noncardiomyocyte gene promoters in the presence and absence of repressor proteins and measuring H3K27me3 deposition.

Our study finds support in the literature. The Chien group (27) in 1994 demonstrated that in heterokaryons with equal numbers of embryonic fibroblast and cardiomyocyte nuclei, cardiomyocyte genes were silenced and there was no expression of cardiomyocyte-specific genes. Gupta *et al.* (28) showed that a palindrome of two Ets-binding sites is important for the cardiomyocyte-restricted expression of the Myh6 (α-myosin heavy chain) gene as deletion of these Ets-binding sites induced Myh6 expression in cells in which the gene is typically silent (28, 29). Subsequent studies found that the repressive actions of the palindromic Ets-binding sites within the Myh6 gene required the proteins PurA and PurB (30).

Ablation of the three repressors was sufficient to induce fibroblasts to convert into cardiomyocyte-like cells *in vivo*. Future studies are needed to determine if the rate of conversion is sufficient to promote significant functional recovery in cardiac injury models. It would also be important to measure the electrophysiological profiles of the cardiomyocytes derived from fibroblasts to determine their similarity to pre-existing cardiomyocytes.

In summary, our data imply that silencing of tissue-specific genes is hierarchal. Sequence-specific proteins such as Cbx1, PurB, and Sp3 bind to the tissue-specific gene. Once bound to the tissue-specific gene, these proteins then act as a scaffold. The scaffold plays two roles. First, to induce a conformational change in the DNA, which acts as a conduit for nucleosome binding. Second, to bring in enzyme complexes such as the PRC2, which mediate long-term gene silencing *via* modifications of the histones within the nucleosome core.

## Experimental procedures

### Cell isolation

Cardiomyocytes and fibroblasts (cardiac, lung, and tail tip) were derived from 1-day-old neonate C57BL6 mice and cultured according to the established protocols (31).

### Human cardiac fibroblasts

Human cardiac fibroblasts were acquired from Cell Applications, Inc (306-05f) and were cultured according to the manufacturer’s instructions.

### Generating iPS-derived cardiomyocytes

Human iPS cells were differentiated into cardiomyocytes according to Burridge *et al.* (32).

### Repressor knockdown

siRNAs were purchased from Qiagen. In the initial screen, four siRNAs (20 μM stock) were used for each repressor. The siRNA that gave rise to the highest level of knockdown was used for future experiments: Cbx1 siRNA 4 (catalog no.: SI00942676), Ddx5 siRNA 2 (catalog no.: S100976514), Egr1 siRNA 1 (catalog no.: S100990899), Fhl2 siRNA 4 (catalog no.: S100190960), Fli1 siRNA 1 (catalog no.: S101003471), PurB siRNA 2 (catalog no.: SI01393462), Sp3 siRNA 2 (catalog no.: SI01429918), Tcf4 siRNA 6 (catalog no.: S102715461), and Zfp36 siRNA 5 (catalog no.: S105451670). A nontargeting siRNA was used as a control (Dharmacon; catalog no.: D-001810-03-05). For transfection, cardiac fibroblasts were seeded into 12-well plates at 22,500 cells per well 1 day prior to transfection. On the day of transfection, siRNAs (0.75 μl) were diluted in serum-free Dulbecco's modified Eagle's medium (American Type Culture Collection; 99.25 μl). In a separate tube, 0.75 μl of Dharmafect-I (Dharmacon) was diluted with 99.25 μl Opti-MEM serum-free media. After 5 min of incubation, the two solutions were combined. After 20 min, complexes were added to cells along with complete media (550 μl), and the transfection complexes were added to the cells. Knockdown was verified 4 days post-transfection. When used in conjunction with miRNA transfection, siRNA and miRNA transfection complexes were set up independently as described and then added to the cells together. When siRNA and miRNA were used in conjunction, the amount of complete media was reduced (250 μl).

### Direct cardiac reprogramming with miR combo

Mouse (C57BL/6) neonatal cardiac fibroblasts were isolated from 2-day-old mouse neonates according to the method outlined in the study by Jayawardena *et al.* (31). Following isolation, fibroblasts were cultured in growth media containing Dulbecco's modified Eagle's medium (American Type Culture Collection; catalog no.: 30-2002) supplemented with 15% v/v fetal bovine serum (FBS; Thermo Fisher Scientific; HyClone FBS; catalog no.: SH30071.03; Lot number: AXK49952) and 1% v/v penicillin/streptomycin (Gibco; catalog no.: 15140-122, 100 units penicillin, 100 μg/ml streptomycin). Fibroblasts were passaged once the cells had reached 70 to 80% confluence using 0.05% w/v trypsin (Gibco; catalog no.: 25300-054). Freshly isolated fibroblasts were labeled as passage 0. Experiments were conducted with cells at passage 2. For all experiments, cells were seeded at 5000 cells/cm^2^ in growth media. After 24 h, the cells were transfected with transfection reagent alone (Dharmafect-I; Thermo Fisher Scientific), with transfection reagent plus nontargeting miRNAs (negmiR), or with transfection reagent plus our previously reported combination of cardiac reprogramming miRNAs (miR combo, miR-1, miR-133, miR-208, miR-499).

### qPCR

Total RNA was extracted using a Quick-RNA MiniPrep Kit according to the manufacturer’s instructions (Zymo Research). Total RNA (50–100 ng) was converted to complementary DNA (cDNA) using a High-Capacity cDNA Reverse Transcription kit (Applied Biosystems). cDNA was used in a standard qPCR involving FAM-conjugated gene-specific primers (Thermo Fisher Scientific) and TaqMan Gene Expression Master Mix (Thermo Fisher Scientific). Primers were acquired from Thermo Fisher Scientific, and the following are the assay ID numbers: Actn2 Mm00473657_m1; Cacna1c Mm00437917_m1; Gapdh Mm99999915_m1; Kcna4 Mm01336166_m1; Kcnh2 Mm00465377_mH; Kcnj2 Mm00434616_m1; Kcnk6 Mm01176312_g1; Myh6 Mm00440359_m1; Myoz2 Mm00469639_m1; Nebl Mm00503886_m1; Ryr2 Mm00465877_m1; Scn5a Mm01342518_m1; Tnnc1 Mm00437111_m1; Tnni1 Mm00502426_m1; Tnni3 Mm00437164_m1; Tnnt2 Mm01290256_m1; and Ttn Mm00621005_m1.

### MNase-Seq and ChIP-Seq

Isolated mouse (C57BL/6) neonatal cardiac fibroblasts (900,000 cells; passage 2) were seeded into T150 flasks in growth media. Where necessary, the next day, cells were transfected with a nontargeting control siRNA or an siRNA targeting Cbx1, PurB, or Sp3 as described previously. Seven days after seeding, chromatin was isolated with a SimpleChIP Plus Enzymatic Chromatin IP Kit (Cell Signaling; catalog no.: 9005) according to the manufacturer’s instructions. Once isolated, chromatin was digested with the supplied MNase (1.5 ml of a 1:10 dilution for 900,000 cells) according to the manufacturer’s instructions. The amount of MNase was empirically determined to digest chromatin to one nucleosome in length. MNase-digested chromatin was then used for MNase-Seq. MNase-digested chromatin was also used for ChIP-Seq. Here, MNase-digested chromatin (900,000 cells) was incubated overnight with 8 μg of control immunoglobulin G, Cbx1 (Cell Signaling; catalog no.: 8676), PurB (Proteintech Group, Inc; catalog no.: 18128-1-AP), or Sp3 (Thermo Fisher Scientific; catalog no.: PA5-78176) antibodies. High-throughput sequencing was performed by the Duke Genomic Core. In total, five independent experiments were performed, and libraries were generated with a NovaSeq 6000 kit (Illumina). Libraries were pooled and run in duplicate (50 bp paired end) with an Illumina NovaSeq 6000. Sequencing depth was >25 × 10^6^ individual reads per sample. Individual bioinformatics programs within the Galaxy suite were used for sequence alignment, peak calling, and peak comparisons. Adaptors were removed, and sequences were then aligned to mouse reference genome mm10 using Bowtie2 (33). For MNase-Seq, bamcoverage was used to determine nucleosome positions with annotated genes broken up into 10 bp bins ± 1 kb around the transcription start site and read counts counted for each bin and normalized to the effective size of the mouse genome. For ChIP-Seq, MACS2 CallPeak (paired-end model) was used to identify peaks with *p* < 0.01. Peaks present in both duplicate samples were identified.

### CRISPR–Cas9 gene editing

Sense and antisense strands for each gRNA (see later) were resuspended in water (100 μM).

The gRNA sequences are as follows:

Cbx1 Sense: ^5′^CACCGCAAAACAAGAAGAAAGTGG^3′^

Cbx1 Antisense: ^5′^AAACCCACTTTCTTCTTGTTTTGC^3′^

PurB Sense: ^5′^CACCATCCGCCAGACGGTGAACCG^3′^

PurB Antisense: ^5′^AAACCGGTTCACCGTCTGGCGGAT^3′^

Sp3 Sense: ^5′^CACCGCTGCCTTGGACGTGGACGG^3′^

Sp3 Antisense: ^5′^AAACCCGTCCACGTCCAAGGCAGC^3′^

Following resuspension, the sense and antisense strands were annealed and phosphorylated. Phosphorylation of the strands (100 nmol per strand) was catalyzed by T4 polynucleotide kinase (New England BioLabs [NEB]). After a 30 min of incubation at 37 °C, the enzyme was inactivated by heating the reaction at 95 °C for 5 min. Annealing of the two strands was then carried out by ramping the temperature down to 25 °C at 5 °C min^−1^. Phosphorylated and annealed gRNAs were then cloned into an pSpCas9(BB)-2A-GFP plasmid (Addgene; plasmid ID: 48138). The following ligation reaction was set up for each gRNA: 100 ng pSpCas9(BB)-2A-GFP, 20 nmol gRNA, 2 μl Tango buffer (Thermo Fisher Scientific), 1 μl 10 mM DTT (Cell Signaling), 1 μl of 10 mM ATP (NEB), 1 μl Bbsl (Thermo Fisher Scientific), and 1 μl T4 DNA ligase (NEB) in a final volume of 20 μl. The ligation reaction was incubated for 1 h at 4 °C. Following ligation, the mixture was used (5 μl) to transform DH5α Competent Cells (NEB) as per the manufacturer’s protocol. Standard overnight DNA minipreps and DNA sequencing were used to ensure correct insertion of the gRNAs.

#### In vitro

3T3 cells were seeded at 5625/cm^2^ in growth media (15% FBS and 1% penicillin/streptomycin). The next day, cells were transfected with 1 μg plasmid DNA using the transfection reagent Lipofectamine 2000 (Thermo Fisher Scientific) as per the manufacturer’s protocol. After 24 h, transfection complexes were removed and replaced with growth media. Three days after transfection, puromycin (2.25 μg/ml; Sigma–Aldrich) was added daily for a total of 7 days to select for transfected cells. Cells were then harvested, and protein was isolated for immunoblotting.

#### In vivo

The lentiviral CRISPR–Cas9 plasmid (1 μg), LentiCRISPRv2 (Addgene; plasmid ID: 52961) was digested for 2 h at 37 °C with 3 μl BsmBI (NEB) in a 60 μl reaction containing FastAP (3 μl; Thermo Fisher Scientific), NEBuffer r3.1 (6 μl; NEB), and 100 mM DTT (0.6 μl; NEB). Digested plasmid was subsequently gel purified using a QIAquick Gel Extraction kit (Qiagen). Following purification, digested plasmid DNA (50 ng) was ligated with the phosphorylated and annealed gRNAs (1 nmol) in a 10 μl reaction containing T4 DNA ligase (1 μl; NEB). Ligations were performed overnight at 4 °C. The next day, ligated products (5 μl) were transformed into DH5α Competent Cells as per the manufacturer’s protocol. Plasmid constructs were verified by restriction digest and DNA sequencing. Lentivirus was generated *via* Lenti-Pac HIV Expression Packaging kit (GeneCopoeia) according to the manufacturer’s instructions. Viral particles (2 × 10^6^ plaque-forming units in 40 μl) were injected into the left ventricle at two sites.

### Images

Images were processed with CorelDraw and Zeiss software (Axiovision Rel 4.8 and Zen Blue).

### Statistics

All statistical analyses were performed using GraphPad (GraphPad Software, Inc). Two-tailed *t* tests were used for studies with two groups. For more than two groups, one-way ANOVAs were used. For ANOVA, Bonferroni post hoc tests were used to determine significance between groups. Individual data points and the mean are shown in all graphs. A *p* value of less than 0.05 was considered significant.

### Study approval

Experiments using animals were approved by the Duke University Division of Laboratory Animals and the Duke Institutional Animal Care and Use Committee.

## Data availability

Raw sequencing data can be found at the Single Read Archive (accession number: SAMN12628632). All other data are contained within the article.

## Supporting information

This article contains supporting information.

## Conflict of interest

V. J. D. and C. P. H. are cofounders of Recardia Therapeutics. This company is focused on developing miRNAs that reprogram fibroblasts into cardiomyocytes. All other authors declare that they have no conflicts of interest with the contents of this article.
