# Supplementary material for: A novel Cbx1, PurB, and Sp3 complex mediates long-term silencing of tissue- and lineage-specific genes
Source: J Biol Chem. 2022 May 20;298(6):102053. doi: 10.1016/j.jbc.2022.102053 (PMC9190063; doi:10.1016/j.jbc.2022.102053)
Supplement: Supplemental Figures S1 and S2 [file mmc1.pdf]

Supporting Information.

Figure S1. Repressor knockdown in lung and tail-tip fibroblasts.

Figure S2. Fig 1D representative graphs.

Table S1. Repressor Binding Genes. Table identifies targets for Cbx1, PurB or Sp3. Cbx1, PurB and Sp3 binding beaks were defined as those >2-fold above background. Genes common to Cbx1, PurB and Sp3 are highlighted in pink.

Table S2. Cbx1 ChIP-seq gene ontology. Gene-ontology for Cbx1 binding genes.

Table S3. PurB ChIP-seq gene ontology. Gene-ontology for PurB binding genes.

Table S4. Sp3 ChIP-seq gene ontology. Gene-ontology for Sp3 binding genes.

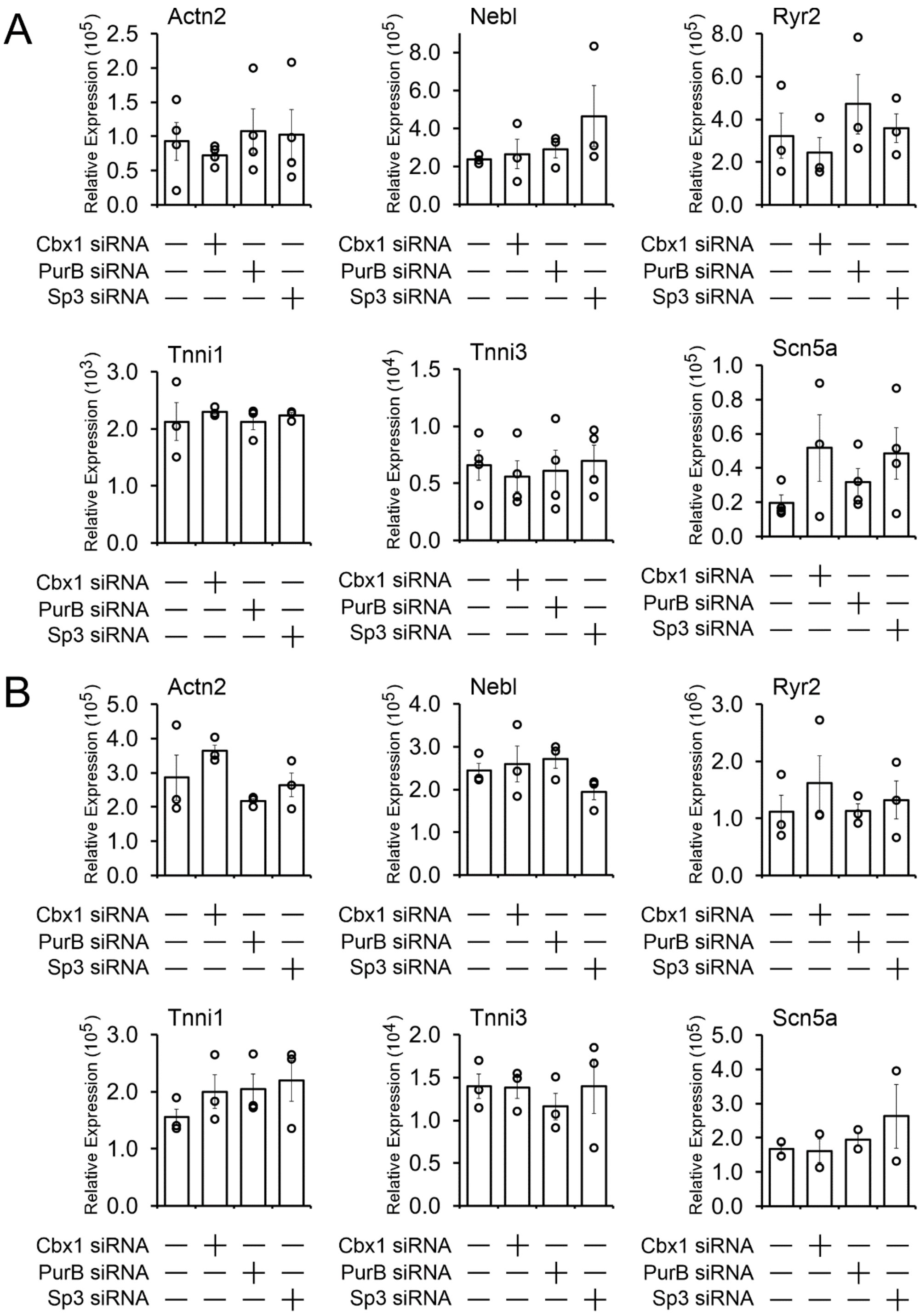

Figure S1. Repressor knockdown in lung and tail-tip fibroblasts. (A) Lung or (B) tail-tip fibroblasts were transfected with a siRNA targeting an individual putative repressor (Cbx1, PurB or Sp3) or a non-targeting siRNA as a control. After 14 days, expression of the indicated cardiomyocyte-specific genes was determined by qPCR. Expression values were normalized to the control siRNA. N=3.

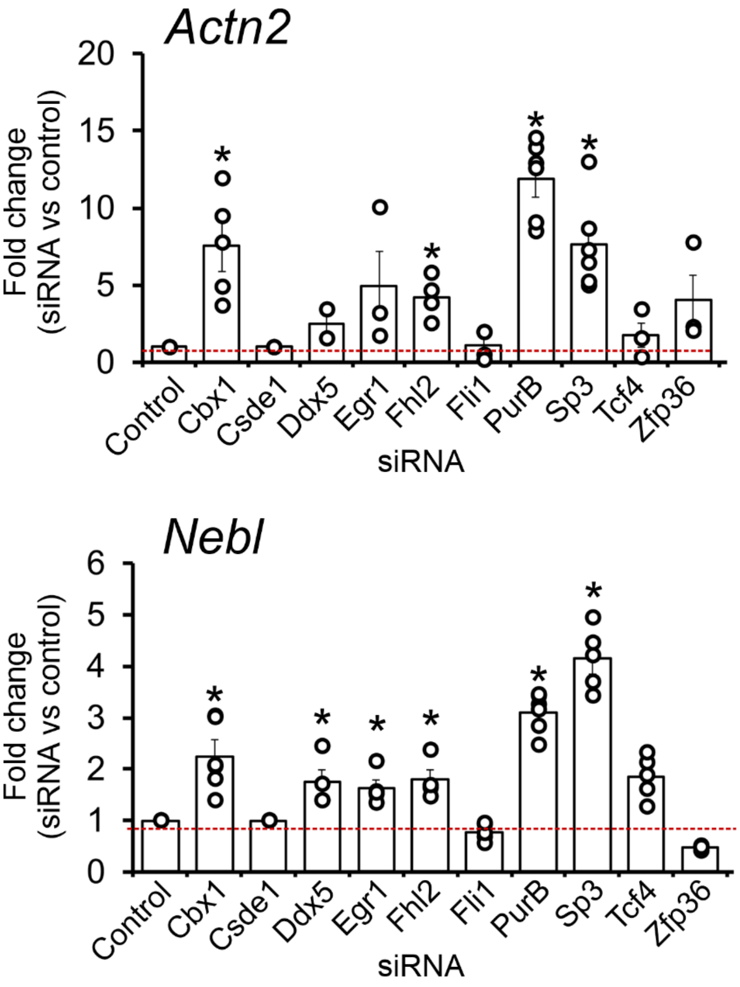

Figure S2. Fig 1D representative graphs. Two example genes, Actn2 and Nebl, from Fig 1D. Expression values were normalized to the control siRNA. N=3-5. *P<0.05.
